# Maternal Adaptive Immune Cells in Decidua Parietalis Display a More Activated and Coinhibitory Phenotype Compared to Decidua Basalis

**DOI:** 10.1155/2017/8010961

**Published:** 2017-11-29

**Authors:** Martin Solders, Laia Gorchs, Sebastian Gidlöf, Eleonor Tiblad, Anna-Carin Lundell, Helen Kaipe

**Affiliations:** ^1^Division of Therapeutic Immunology, Department of Laboratory Medicine, Karolinska Institutet, Karolinska University Hospital, 14186 Stockholm, Sweden; ^2^Center for Fetal Medicine, Karolinska University Hospital, Huddinge, 141 86 Stockholm, Sweden; ^3^Department of Clinical Science, Intervention and Technology, Karolinska Institutet, Karolinska University Hospital, Huddinge, 141 86 Stockholm, Sweden; ^4^Department of Women's and Children's Health, Karolinska Institutet, 171 77 Stockholm, Sweden; ^5^Department of Rheumatology and Inflammation Research, Sahlgrenska Academy, University of Gothenburg, Gothenburg, Sweden; ^6^Department of Clinical Immunology and Transfusion Medicine, Karolinska University Hospital, Huddinge, 141 86 Stockholm, Sweden

## Abstract

The maternal part of the placenta, the decidua, consists of maternal immune cells, decidual stromal cells, and extravillous fetal trophoblasts. In a successful pregnancy, these cell compartments interact to provide an intricate balance between fetal tolerance and antimicrobial defense. These processes are still poorly characterized in the two anatomically different decidual tissues, basalis and parietalis. We examined immune cells from decidua basalis and parietalis from term placentas (*n* = 15) with flow cytometry. By using multivariate discriminant analysis, we found a clear separation between the two decidual compartments based on the 81 investigated parameters. Decidua parietalis lymphocytes displayed a more activated phenotype with a higher expression of coinhibitory markers than those isolated from basalis and contained higher frequencies of T regulatory cells. Decidua basalis contained higher proportions of monocytes, B cells, and mucosal-associated invariant T (MAIT) cells. The basalis B cells were more immature, and parietalis MAIT cells showed a more activated phenotype. Conventional T cells, NK cells, and MAIT cells from both compartments potently responded with the production of interferon-*γ* and/or cytotoxic molecules in response to stimulation. To conclude, leukocytes in decidua basalis and parietalis displayed remarkable phenotypic disparities, indicating that the corresponding stromal microenvironments provide different immunoregulatory signals.

## 1. Introduction

During pregnancy, the fetus receives nutrients, gas exchange, and immunological protection against infections from the mother via the placenta. At the same time, the maternal immune system must be kept from attacking the allogeneic fetus. The fetus, umbilical cord (UC), and placenta are encased by the decidua, a maternal membrane originating from differentiated endometrial cells in early pregnancy [1]. The decidua can be divided into two anatomically different parts; the decidua basalis covers the basal plate of the placenta, while the decidua parietalis lines the fetal membranes. Decidual stromal cells (DSCs) make up the foundation of the connective structures of both decidua basalis and parietalis and have been shown to utilize a specific epigenetic program of gene silencing in order to minimize the attraction of maternal effector T cells in mice [2]. *In vitro*, DSCs have been shown to induce T regulatory cells as well as suppress T cell proliferation by utilizing IDO and PGE_2_ and mediate an induced impaired ability of T cells to respond to IL-2 [3–5]. Trophoblast cells make up the outer barrier of the fetal side of the placenta, and over the course of pregnancy, trophoblasts invade the decidual layer surrounding the placenta. These extravillous trophoblasts lack classical polymorphic HLA class I molecules but express HLA-C, HLA-G, and HLA-E [6]. This provides some aspects of how the fetus escapes immunological recognition and response.

Previous studies on different subsets of maternal immune cells infiltrating decidual tissues rarely discern between decidua parietalis and basalis. In early pregnancy, the majority of decidual leukocytes are noncytotoxic NK cells, which have been shown to be crucial in the forming and remodeling of the blood supply to placental tissues. Decidual macrophages have an important role in maintaining a tolerogenic environment in healthy pregnancies, but can also respond with proinflammatory cytokines upon bacterial stimulation [7]. The decidual macrophage population also contains a subclass with an M2 phenotype, which produces high levels of IL-10 [8]. The number of T cells increases over the course of pregnancy and constitutes between 30 and 80% of the total lymphocytes in the decidua at term [9]. The number of T regulatory cells is elevated in both decidual compartments and has the ability to suppress immune responses against the fetus [10]. The expression of PD-1 and TIM-3 is greatly increased on decidual T cells, and *in vivo* blockade of these molecules in mice results in increased miscarriage rates [11].

As placental tissues have emerged as a promising source of stem cells for clinical trials [12], it is of importance to characterize the physiological state of the surrounding immune cell populations in this compartment. The factors influencing immune cell composition and activation status in the decidua basalis and parietalis are still poorly characterized, but it is likely that these two sites are differentially influenced by the stromal microenvironment.

The aim of this study was to examine the immune cell composition of these two decidual tissues. Using flow cytometry, we have made an in-depth characterization of lymphocyte populations in the different decidual compartments from term placentas donated after uncomplicated pregnancies. This provides new basic knowledge of the immunological landscape in these tissues, as well as potential insights into how the stromal environment in different decidual sites can mediate immune regulation.

## 2. Material and Methods

### 2.1. Placental Donors

Following uncomplicated term pregnancies (median gestation week 39, range 38–42), healthy individuals (*n* = 15, median age 32, range 21–40) donated their placentas following elective caesarian sections. Written informed consent was obtained from the donors, and the regional review board of ethics in research of Karolinska Institutet approved the donation of peripheral blood and placentas (entry numbers 2009/418-31/4, 2010/2061-32, and 2015/1848-31/2). Data on some immune parameters in decidua parietalis from 11 out of 15 donors have partly been included in another publication [13], but no data on the decidua basalis immune cells has previously been published.

### 2.2. Cell Isolation

Placentas were transported straight to our laboratory from the operating room in the adjacent building, and the cell isolation started in less than 30 minutes following the placental delivery. Paired samples of tissue-resident lymphocytes were collected from decidua basalis and parietalis using a method similar to that used by others [14]. The fetal membranes (including the decidua parietalis) were cut ≥1 cm from the edge of the placenta and placed in a sterile petri dish and washed extensively with PBS. The parietalis was dissected from the chorion, which was then discarded together with the amnion. The tissue was cut into smaller pieces and placed in PBS. Thereafter, the placenta was placed with the umbilical cord facing down and washed extensively with PBS. The basalis is strongly attached to the placental tissue, and hence we used a scalpel to carefully scrape off the thin grey basalis membrane which was placed in PBS. The two types of tissue were washed in PBS by centrifugations at 600*g* for 1 minute. The supernatant was discarded, and the process was repeated five times or more until the supernatant was clear. Lymphocytes were released from the tissue by nonenzymatic mechanical disaggregation using the gentleMACS Dissociator (Miltenyi Biotec, Bergisch Gladbach, Germany). The tissue was then consecutively filtered through a 100 *μ*m metal mesh and then through 70 *μ*m and 40 *μ*m cell strainers (VWR, Radnor, PA). Following centrifugation, the cell pellets were resuspended in lysis buffer (IOTest 3, Beckman Coulter, Fullerton, CA, USA), washed once, and stained for extracellular flow cytometric analysis. The remaining cells were frozen in liquid nitrogen in RPMI (HyClone, GE Health Sciences, South Logan, UT) medium supplemented with 10% fetal calf serum, 100 U/ml penicillin, and 100 *μ*g/ml streptomycin (complete medium) containing 10% DMSO.

### 2.3. Flow Cytometry

The majority of the extracellular flow cytometry experiments were performed directly on fresh cells, but for six donors, the T cell markers CD69, CD25, CXCR3, CCR6, CD38, HLA-DR, CD127, and PD-1 were analyzed on paired frozen samples. Staining was carried out in 96-well plates with ≤1 × 10^6^ cells/well in 50 *μ*l CliniMACS PBS/EDTA buffer (Miltenyi Biotech, Bergish Gladbach, Germany) supplemented with 0.1% bovine serum albumin. The cells were incubated with mAbs for 30 min at 4°C. Intracellular staining was performed after extracellular staining using the BD Cytofix/Cytoperm™ kit (BD Biosciences, Franklin Lakes, NJ) according to the manufacturer's instructions. 7AAD staining was used to sort live and dead cells if no intracellular staining was performed. The antibodies used in this study are listed in Supplementary Table 1 available online at https://doi.org/10.1155/2017/8010961. Data was collected using a BD FACSCanto flow cytometer and analyzed with FlowJo software (Tree Star, Ashland, OR, USA). Results from subgating were only included if the parent population consisted of ≥80 cells.

### 2.4. Bacterial Stimulation Assay

Mononuclear cells were isolated by density gradient centrifugation (Lymphoprep, Axix-Shield, Dundee, Scotland) and resuspended in complete medium. Fresh cells were then cultured at 37°C and 5% CO_2_, at a concentration of 3 × 10^6^ cells/ml, in 96-well plates alone or together with UV-irradiated *Escherichia coli* (*E. coli*) at a multiplicity of infection of 30 together with 1.25 *μ*g/ml anti-CD28mAb (CD28.2, BioLegend, San Diego, CA). After 12 hours of culture, 10 *μ*g/ml Brefeldin-A (BFA, Sigma-Aldrich, St. Louis, MO) was added, followed by an additional 4 hours of culture. After the total 16 hours of culture, cells were harvested and stained for flow cytometry.

### 2.5. PMA/Ionomycin Stimulation Assay

Frozen cells were thawed and mononuclear cells were isolated by density gradient centrifugation (Lymphoprep, Axix-Shield). Peripheral blood mononuclear cells (PBMCs) from healthy blood donors (healthy controls) were used as controls. Cells were resuspended in complete medium and cultured at 37°C and 5% CO_2_, at a concentration of 2 × 10^6^ cells/ml in 96-well plates. 10 *μ*g/ml BFA (Sigma-Aldrich) was added to all wells, and half of the samples were stimulated with 25 ng/ml PMA (Sigma-Aldrich) and 1 *μ*g/ml ionomycin (Sigma-Aldrich). After 5 hours of culture, cells were harvested and stained for flow cytometry.

### 2.6. Statistics

**Figure 1:**
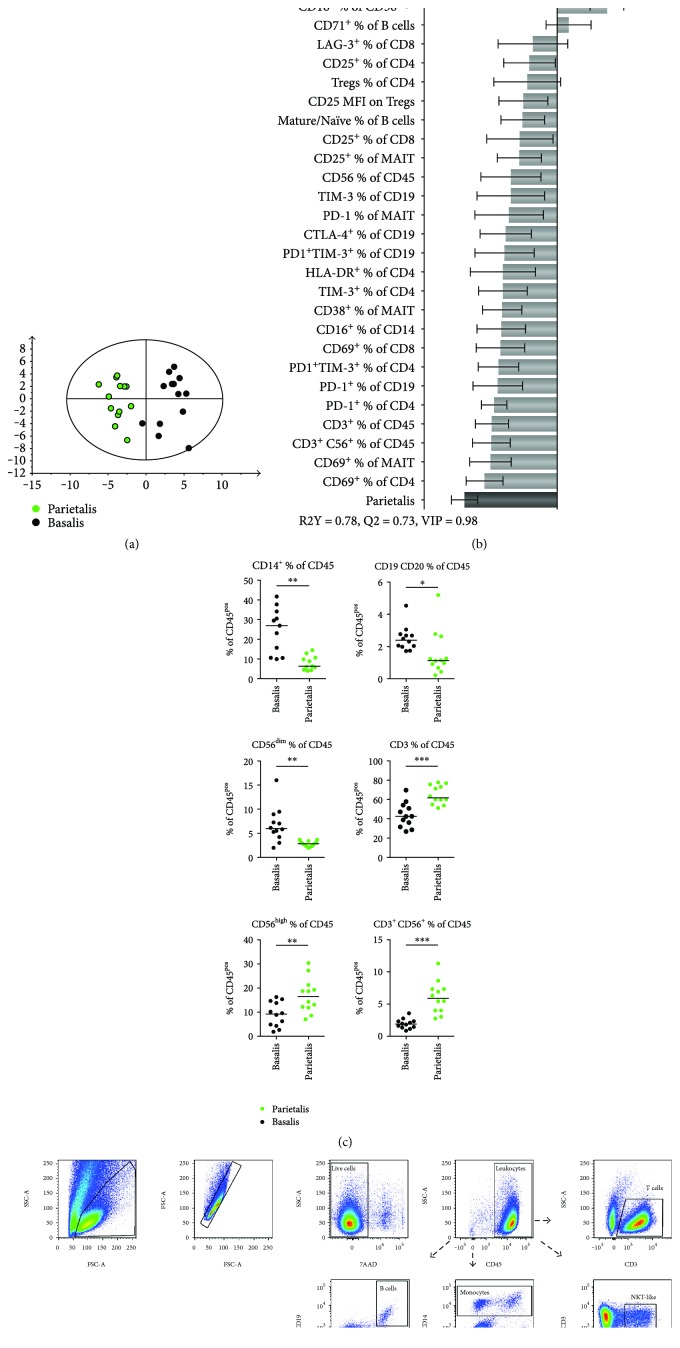
Different leukocyte populations in decidua parietalis and basalis. (a) OPLS-DA observation plot displaying a separation between leukocytes from decidua basalis and parietalis. (b) OPLS plot following a variable influence on projection (VIP) of 0.98, showing associations between decidual compartment and phenotypic leukocyte markers (*n* = 8–13 for (a, b)). (c) Distribution of major leukocyte subsets in paired samples of decidua basalis and parietalis compared with the nonparametric Wilcoxon test. Line in graphs depicts the median among values (*n* = 11-12). (d) Representative flow cytometry plots showing the initial gating strategy used throughout the paper, as well as the gating of the subsets in (c). ^∗^*p* < 0.05; ^∗∗^*p* < 0.01; ^∗∗∗^*p* < 0.001.

**Figure 2:**
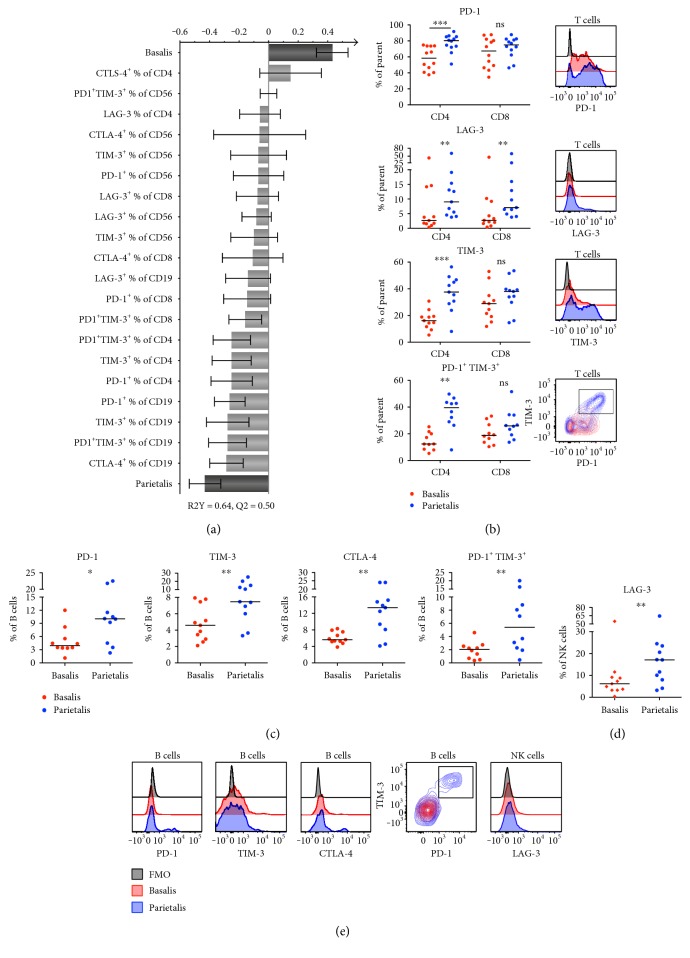
Lymphocytes in decidua parietalis express more coinhibitory markers compared to basalis. (a) OPLS plot showing associations between decidual compartment and phenotypic coinhibitory markers (*n* = 10–12). (b) Surface expression and representative histograms and contour plots showing the expression of the indicated extracellular markers on decidua basalis and parietalis T cells (CD3^+^) compared to the fluorescent minus one (FMO) control of PD-1 (*n* = 12), LAG-3 (*n* = 11), TIM-3 (*n* = 11), and PD-1^+^TIM-3^+^ (*n* = 10). (c) B cell (CD19^+^) surface expression of PD-1 (*n* = 10), TIM-3 (*n* = 11), CTLA-4 (*n* = 11), and PD-1^+^TIM-3^+^ (*n* = 10). (d) NK cell (CD56^+^CD3^−^) surface expression of LAG-3 (*n* = 11). (e) Representative histograms and contour plots showing the expression of the indicated extracellular markers on decidua basalis and parietalis cells from (c) to (d) compared to the fluorescent minus one (FMO) control. Line in graphs depicts the median. Comparisons between the paired samples were made using the nonparametric Wilcoxon test. ns = not significant; ^∗^*p* < 0.05; ^∗∗^*p* < 0.01; ^∗∗∗^*p* < 0.001.

**Figure 3:**
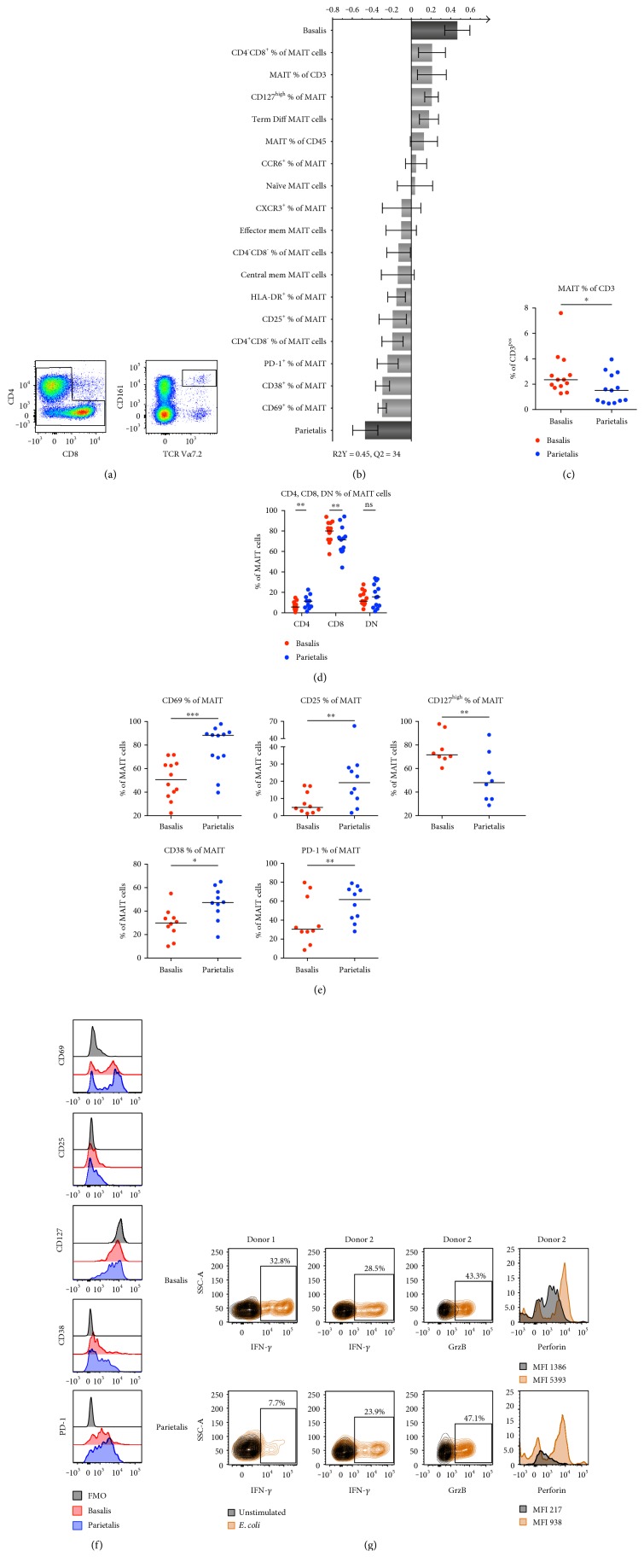
MAIT cells in decidua basalis are more numerous, but parietalis MAIT cells display a more activated phenotype. (a) Representative flow cytometry plots showing the gating strategy for MAIT cells based on CD3^+^ T cells (left to right). (b) OPLS plot showing associations between decidual compartment and phenotypic MAIT cell markers (*n* = 8–13). (c) Number of MAIT cells expressed as percentage of CD3^+^ T cells in paired samples of decidua basalis and parietalis (*n* = 13). (d) MAIT cell surface expression of CD4 and CD8 in paired samples of decidua basalis and parietalis (*n* = 13). (e) MAIT cell surface expression of CD69 (*n* = 12), CD25 (*n* = 10), CD127^high^ (*n* = 8), CD38 (*n* = 10), and PD-1 (*n* = 10). (f) Representative histograms showing the expression of the indicated extracellular markers on MAIT cells from decidua basalis and parietalis compared to the fluorescent minus one (FMO) control. (g) Contour plots and histograms showing the expression of the indicated intracellular molecules on decidua basalis (top) and parietalis (bottom) in unstimulated (black) compared to the *E. coli* stimulated conditions (orange) for interferon-*γ* (IFN-*γ*, *n* = 2), granzyme B (GrzB, *n* = 1), and perforin (*n* = 1). Line in graphs depicts the median. Comparisons between the paired samples were made using the nonparametric Wilcoxon test. ns = not significant; ^∗^*p* < 0.05; ^∗∗^*p* < 0.01; ^∗∗∗^*p* < 0.001.

Multivariate orthogonal projection to latent structures by means of partial least squares discriminant analysis (OPLS-DA) was used to obtain a maximum separation of *X*-variables, that is, immune cell variables, based on class information, that is, basalis and parietalis in Figures 1(b), 2(a), and 3(b) (SIMCA software, Sartorius Stedim Biotech, Umeå, Sweden). The contribution of each *X*-variable, VIP values, to the OPLS-DA model in Figure 1(a) was calculated (Supplementary Figure S1). *X*-variables with a VIP value below 0.98 were excluded, and a new model was generated based on remaining variables (Figure 1(b)). The scale presented on the *y*-axis of the OPLS plot is a dimensionless scale; the loading vector is normalized to unit length. The quality of OPLS analyses is based on R2, which indicates how well the variation of the variables is explained by the model, and Q2, an estimate of the model's predictive ability. Utilizing the OPLS-DA analysis as screening for differences between the groups, the factors contributing most to the separation were further analyzed using a two-tailed Wilcoxon matched-pairs signed rank test (GraphPad Software, La Jolla, CA). An alpha value of <0.05 was considered significant.

## 3. Results

### 3.1. The Proportions of Several Immune Cell Populations Differ in Decidua Basalis and Parietalis

By multivariate OPLS-DA, we examined if the 81 immunological immune cell variables assessed differed between decidua basalis and parietalis. As depicted in the observational plot in Figure 1(a), a clear separation between decidua basalis and parietalis was found. The *X*-variables that showed the strongest association with basalis or parietalis are shown in the OPLS-DA loading plot in Figure 1(b), a model based on *X*-variables with VIP values ≥ 0.98. A VIP column plot for all parameters is shown in Supplementary Figure S1. Further investigating the two groups using univariate statistical analysis, differences were seen in all main immune cell populations investigated. The proportions of monocytes, B cells, and CD56^dim^ NK cells were higher in basalis compared to parietalis, whereas total CD3^+^ T cells, CD56^bright^ NK cells, and NKT-like cells were found in higher frequencies in parietalis relative to basalis (Figure 1(c), gating strategies in Figure 1(d)).

### 3.2. Decidua Parietalis T Cells Display a More Activated Phenotype Compared to Decidua Basalis

**Figure 4:**
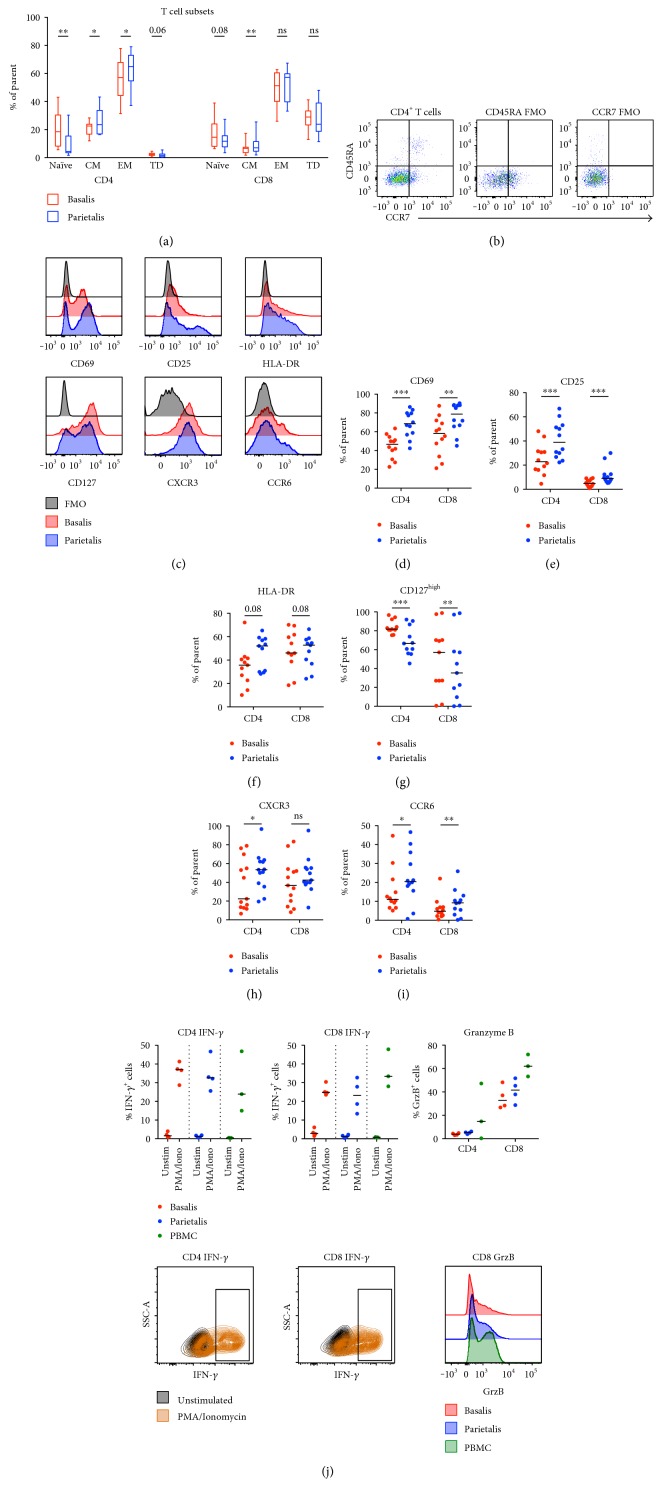
Decidua parietalis contains more activated T cells than basalis. (a) CD4^+^ and CD8^+^ T cells were gated according to the expression of CD45RA and CCR7 (gating strategy in (b)) and divided into naïve, central memory (CM), effector memory (EM), or terminally differentiated (TD) subsets (*n* = 13). (c) Representative histograms showing the expression of the indicated extracellular markers on decidua basalis and parietalis compared to the fluorescent minus one (FMO) control. Comparisons between decidua basalis and parietalis CD4^+^ and CD8^+^ T cells regarding the expression of CD69 (d), CD25 (e), HLA-DR (f), CD127^high^ (g), CXCR3 (h), and CCR6 (i) ((d, e) and (i) *n* = 12, (f, g) *n* = 11, (h) *n* = 13). (j) Paired mononuclear cells from basalis (*n* = 4) and parietalis (*n* = 4) were stimulated for 5 hours with PMA/ionomycin or left unstimulated. Data on cells from healthy controls (PBMC) were included as controls (*n* = 3). Intracellular expression of interferon-*γ* (IFN-*γ*) and granzyme B (GrzB) was determined by flow cytometry. T cells were divided into CD4^+^ (upper left) and CD8^+^ (upper middle), representative contour plots below the respective cell subset. GrzB was measured in unstimulated samples only (upper right), representative histograms shown in the bottom right corner. Line in graphs depicts the median among values. Comparisons between the paired samples were made using the nonparametric Wilcoxon test. ns = not significant; ^∗^*p* < 0.05; ^∗∗^*p* < 0.01; ^∗∗∗^*p* < 0.001.

As demonstrated by the OPLS-DA in Figure 1(b), phenotypic differences in T cells, B cells, and NK cells were revealed between decidua basalis and parietalis. Based on CCR7 and CD45RA expression, we found that CD4^+^ and CD8^+^ T cells from both compartments were dominated by an effector memory T cell phenotype at the expense of naïve cells (Figure 4(a)). This contrasts to the general composition in peripheral T cells in pregnant and nonpregnant women [13, 15]. Decidua parietalis contained a higher proportion of both effector memory and central memory CD4^+^ T cells compared to decidua basalis (Figure 4(a)). Gating strategies are shown in Figure 4(b). Markers of T cell activation and homing also had different expression pattern between the two compartments (Figures 4(c), 4(d), 4(e), 4(f), 4(g), 4(h), and 4(i)). T cells from parietalis had a higher expression of the early activation and/or tissue residency marker CD69 (Figure 4(d)), as well as an increased expression of the *α*-chain of the IL-2 receptor, CD25 (Figure 4(e)). No significant difference was observed for the expression of the late activation marker HLA-DR, although there was a trend towards an increased expression on T cells from parietalis (Figure 4(f)). The expression of the *α*-chain of the IL-7 receptor, CD127, was significantly decreased in parietalis, further indicating a more activated phenotype compared to the T cells from basalis (Figure 4(g)). The expression of the chemokine receptors CXCR3 and CCR6 was both increased in parietalis compared to basalis (Figures 4(h) and 4(i)).

Upon stimulation with PMA/ionomycin, both CD4^+^ and CD8^+^ T cells responded with the production of interferon-*γ* (IFN-*γ*) in similar levels to that of PBMCs from healthy donors (Figure 4(j)). Decidual CD8^+^ T cells expressed granzyme B (GrzB) in a resting state, but the median expression was lower compared to peripheral T cells from healthy donors (Figure 4(j)). As expected, the expression of GrzB was low in CD4^+^ T cells.

### 3.3. Differences in B Cell and NK Cell Subsets and T Regulatory Cells in Decidua Parietalis and Basalis

**Figure 5:**
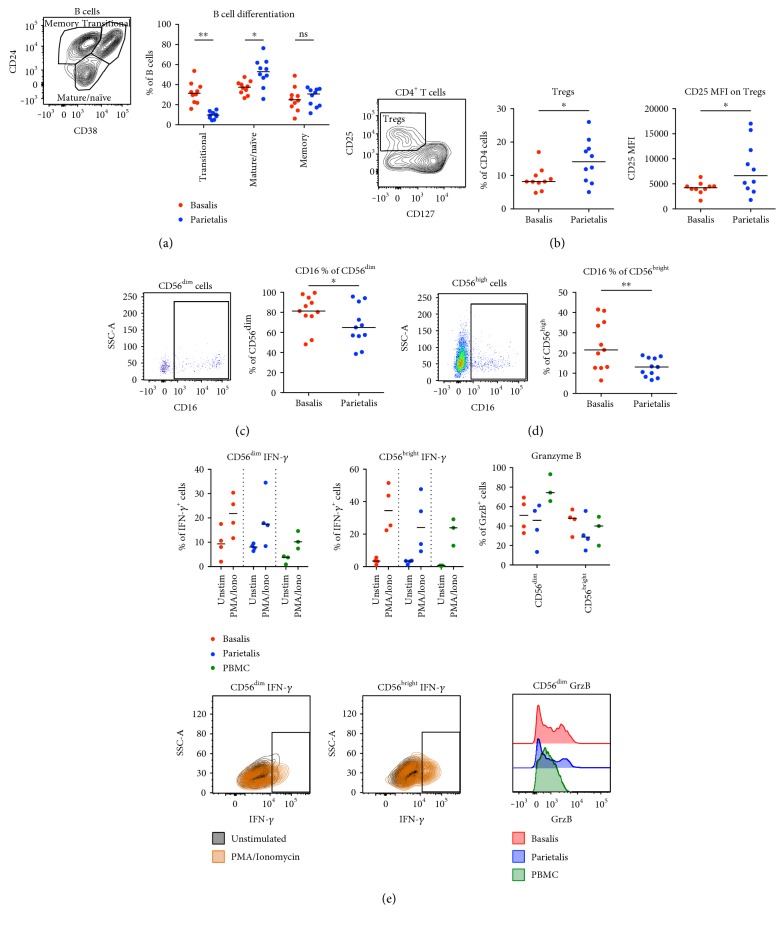
Maturational state of B cells and frequencies of T regulatory cell differ between decidual compartment. (a) Gating strategy (left) and graphical representation (right) of differentiation status of B cells from paired samples of decidua basalis and parietalis (*n* = 10). (b) Gating strategy and proportions of T regulatory cells (Tregs) as percentage of CD4^+^ T cells (middle) and the median fluorescence intensity (MFI) of CD25 of the gated Tregs (right) (*n* = 10). (c) Gating strategy (left) and graphical representation (right) of CD16 expression on CD56^dim^ cells from paired samples of decidua basalis and parietalis (*n* = 11). (d) Same as in (c) but for CD56^high^ cells (*n* = 11). (e) Paired mononuclear cells from basalis (*n* = 4) and parietalis (*n* = 4) were stimulated for 5 hours with PMA/ionomycin or left unstimulated. Data on cells from healthy controls (PBMC) were included as controls (*n* = 3). Intracellular expression of interferon-*γ* (IFN-*γ*) and granzyme B (GrzB) was determined by flow cytometry. T cells were divided into CD56^dim^ (upper left) and CD56^bright^ (upper middle), representative contour plots below the respective cell subset. GrzB was measured in unstimulated samples only (upper right), representative histograms shown in the bottom right corner. Line in graphs depicts the median. Comparisons between the paired samples were made using the nonparametric Wilcoxon test. ns = not significant; ^∗^*p* < 0.05; ^∗∗^*p* < 0.01.

B cells can be divided into immature/transitional, mature/naïve, and memory cells based on CD24 and CD38 expression (Figure 5(a)). The frequency of transitional B cells (CD24^high^CD38^high^) among CD19^+^CD20^+^ B cells was higher in basalis compared to parietalis, whereas the mature/naïve compartment (CD24^int^CD38^int^) was higher in parietalis (Figure 5(a)). There was no significant difference in the B cell memory compartment (CD24^high^CD38^low/neg^).

It has previously been shown that CD4^+^CD25^+^CD127^low^ T cells in decidual tissues largely overlap with Foxp3^+^ T regulatory cells (Tregs) and that they are increased in the first trimester compared to peripheral blood [16]. We found that parietalis contained a significantly higher proportion of CD4^+^CD25^+^CD127^low^ Tregs among the CD4^+^ T cells compared to basalis and that the median intensity of CD25 expression in Tregs was higher in parietalis (Figure 5(b)).

The expression of the Fc receptor CD16 was investigated on the two NK cell populations residing in decidual tissue. As expected, the classical CD3^−^CD56^dim^ NK cells had a high CD16 expression (Figure 5(c)), whereas the expression on the decidual CD3^−^CD56^bright^ NK cells was low (Figure 5(d)). The CD16 expression was significantly lower on both NK cell populations in parietalis compared to basalis (Figures 5(c) and 5(d)). On the other hand, there was a trend towards a lower expression of CD16 on monocytes in parietalis compared to basalis (Supplementary Figure S2a).

After stimulation with PMA/ionomycin, we observed that IFN-*γ* was expressed in the CD56^dim^, as well as the CD56^bright^ NK cells from both basalis and parietalis (Figure 5(e)). The proportion of NK cells from decidua expressing IFN-*γ* was as least as high as peripheral NK cells from healthy donors. We further noted that CD56^dim^ NK cells showed a pattern of background expression of IFN-*γ*, which was not observed in CD56^bright^ cells. Although too few experiments were performed to do statistical analysis, the proportion of CD56^dim^ NK cells expressing GrzB appeared to be lower in decidua compared to peripheral blood (Figure 5(e)). However, there was no such difference in GrzB expression in CD56^bright^ NK cells.

### 3.4. Decidua Parietalis Immune Cells Express Higher Levels of Coinhibitory Markers Compared to Basalis

The surface expression of the coinhibitory molecules PD-1, TIM-3, LAG-3, and CTLA-4 was assayed on CD4^+^ and CD8^+^ T cells, CD19^+^ B cells, and CD56^+^ NK cells. Based on these 20 parameters from the flow cytometry results, the OPLS-DA model demonstrated that there were higher proportions of cells expressing coinhibitory molecules, mainly B cells and CD4^+^ T cells, in parietalis compared to basalis (Figure 2(a)). Further analysis with univariate statistics in large confirmed this model. Expression of PD-1, LAG-3, and TIM-3 and coexpression of both PD-1 and TIM-3 were higher in parietalis compared to basalis CD4^+^ T cells (Figure 2(b)). The same results were seen regarding LAG-3 expression in CD8^+^ T cells, but only trends were apparent for the other markers (Figure 2(b)). The expression of CTLA-4 on T cells was not statistically different between basalis and parietalis (Supplementary Figure S2b). An increased frequency of B cells from parietalis expressed PD-1, TIM-3, and CTLA-4, as well as the dual expression of PD-1 and TIM-3 (Figures 2(c) and 2(e)). Regarding the NK cells, the expression of LAG-3 was higher in samples from parietalis compared to basalis (Figures 2(d) and 2(e)). Although not significantly different, we also noted a trend towards a higher median expression of TIM-3 on NK cells in parietalis (67%) compared to basalis (51%, *p* = 0.0537, Supplementary Figure S2c).

### 3.5. Mucosal-Associated Invariant T (MAIT) Cell Proportion and Phenotype Differs in Decidual Basalis and Parietalis

MAIT cells can rapidly respond with secretion of proinflammatory cytokines and cytotoxic molecules when stimulated by microbial-derived vitamin B metabolites in an MHC-related molecule 1- (MR1-) dependent manner [17], but little is known about their importance in pregnancy. MAIT cells were identified as V*α*7.2^+^ and CD161^high^ T cells (Figure 3(a)). OPLS-DA demonstrated a separation between decidua basalis and parietalis based on MAIT cell proportion and phenotype (Figure 3(b)). The frequency of MAIT cells among CD3^+^ T cells was higher in basalis compared to parietalis (Figure 3(c)). The distribution of CD8^+^, double negative (DN), and CD4^+^ MAIT cells was similar as previously reported in peripheral blood [17], but the proportion of CD4^+^ MAIT cells was higher in parietalis compared to basalis at the expense of the CD8^+^ MAIT cells (Figure 3(d)). Similar to what we observed for the conventional T cells, the MAIT cells from parietalis had a higher expression of CD69 and CD25 than basalis and a lower expression of CD127 (Figures 3(e) and 3(f)). CD38, another marker for MAIT cell activation [18] and the coinhibitory marker PD-1, was also increased on parietalis MAIT cells as compared to basalis (Figures 3(e) and 3(f)). Thus, decidua parietalis MAIT cells showed a more activated phenotype compared to basalis MAIT cells.

To examine the functionality of MAIT cells in decidual tissue, mononuclear cells were stimulated with UV-irradiated *E. coli* for 16 hours. The response to the bacteria was measured by intracellular expression of IFN-*γ* (two donors) and GrzB and perforin (one donor). A substantial proportion of the decidual MAIT cells from both donors responded with IFN-*γ* expression (Figure 3(g)). Bacterial stimulation also induced expression of the cytotoxic molecules GrzB and perforin in decidual MAIT cells (Figure 3(g)).

## 4. Discussion

This study provides the most comprehensive mapping of immune cell subsets in decidua parietalis and basalis to date. By analyzing such a large number of factors using paired samples, we can present a multifaceted overview of how the immune system is polarized in the different decidual compartments at term pregnancies. Using OPLS-DA to create a model on which further univariate testing is based provides a more objective basis of statistical analysis as all parameters are used and weighed depending on their relative addition to the model. The discriminate analysis revealed large differences in the immune cell composition between decidua parietalis and basalis.

Among the general leukocyte populations, decidua basalis contained more B cells, monocytes, and CD56^dim^ NK cells, whereas parietalis was comprised of more T cells, NKT-like cells, and CD56^bright^ NK cells. This pattern is in general in line with previous publications examining decidual immune cell subsets [19–22].

Decidual NK cells constitute >70% of decidual leukocytes during the first trimester and are important for directing the invasion of trophoblasts into the decidua by cytokine and chemokine gradients, as well as in the growth and remodeling of spiral arteries. Over the course of pregnancy, these numbers decline to reach similar levels at term as the endometrium of nonpregnant women [23]. Bartmann et al. found that CD16 expression on decidual NK cells in basalis increased over the course of pregnancy [21]. We observed that CD16 expression was significantly higher in basalis compared to parietalis on both CD56^dim^ and CD56^bright^ NK cells. It is not known if the decidual NK cells upregulate the expression of CD16 expression during pregnancy or if there is an infiltration of CD16^+^ NK cells as pregnancy proceeds. Both NK cell subsets produced IFN-*γ* when stimulated with PMA/ionomycin, but the CD56^dim^ cells had a background expression, whereas the CD56^bright^ cells did not. It would be of interest to further study decidual NK cells and their responsiveness to physiological stimulus.

The decidual T cell population was polarized towards an effector memory phenotype at the expense of naïve T cells, a finding most prominent in parietalis. This is in line with the findings of Sindram-Trujillo et al., as well as the consecutive work of Tilburgs et al., who showed an enrichment of effector memory T cells in decidual tissues compared to peripheral blood, also with the most prominent changes seen in parietalis [20, 24]. These effector cells contained large amounts of mRNA coding for cytotoxic molecules, but the translation into functional proteins was impaired. In line with these findings, we observed a trend towards T cells from both basalis and parietalis having lower amounts of GrzB than T cells from peripheral T cells from healthy controls. An elevated activation status of T cells in decidual tissues has also been demonstrated previously [20]. Our data confirm these findings, and the observation that CD127 expression is decreased in decidual T cells further supports an effector memory status and activated phenotype [25].

The expression of the chemokine receptors CXCR3 and CCR6 was higher on T cells from decidua parietalis compared to basalis. This probably reflects the findings of a dominant effector memory phenotype but could also imply a higher proportion of Th1 cells and Th17 cells [26, 27]. DSCs have been shown to have an impaired production of the CXCR3 ligands CXCL9 and CXCL10 [2], but extravillous trophoblasts infiltrating decidual tissue constitutively produce CXCL10 [28]. Svensson-Arvelund et al. further showed a high production of the CCR6 ligand CCL20 by cells from placental tissue explants [28]. CCR6^+^ T cells in the 1st trimester decidua were shown to be fewer compared to matched peripheral blood, indicative of a decreased Th17 polarization during early pregnancy [16]. On the other hand, Feyaerts et al. showed that decidua parietalis contained higher proportions of IFN-*γ* and IL-17 producing T cells compared to peripheral and endometrial T cells [15]. This, together with our findings, indicates that the relative frequency of Th1 cells and Th17 cells increases in parietalis over the course of pregnancy, further promoting the activated T cell profile in this compartment.

Although B cells have generally been ignored in the context of decidual immunology, their presence has been demonstrated in previous publications both by flow cytometry [20, 22, 29, 30] and by immunohistochemistry [31]. We have previously shown that DSCs from both decidua basalis and parietalis readily produce B cell-activating factor of the tumor necrosis factor family (BAFF) after stimulation with interferons [22], suggesting a role for B cell maturation in the decidua. We found that the frequency of B cells was higher in basalis compared to parietalis, but that parietalis contained a higher proportion of mature/naïve B cells at the expanse of transitional B cells. This may indicate that microenvironmental factors in parietalis, including BAFF, induce decidual B cell maturation. Interestingly, it was recently shown that an altered B cell composition in decidua and higher BAFF expression were associated with preterm labor [31].

Tregs have been proposed to play an important role in fetal-maternal tolerance and have been investigated in decidual tissues by several others [14, 30, 32–34]. Tilburgs et al. demonstrated a correlation between HLA-C mismatch between mother and fetus and proportions of both activated CD4^+^CD25^dim^ T cells and CD4^+^CD25^high^ putative Tregs in decidua parietalis [35]. This indicates that fetal alloantigens in the decidua promote maternal T cell activation and also induction of Tregs. In line with the increased activation status of T cells in parietalis, we found that the proportion of CD4^+^CD25^+^CD127^low^ Tregs was higher in parietalis than in basalis. The intensity of CD25 expression on Tregs was also higher in parietalis, further indicating a more activated Treg phenotype. We have previously shown that DSCs from parietalis promoted elevated expression of CD25 on alloantigen-stimulated T cells, which partly was explained by an induction of Tregs [4] and also by elevated IL-2 levels in the supernatant due to a diminished capacity of the T cells to signal through the IL-2 receptor complex [5]. Thus, DSCs could potentially have a role in shaping the T cell phenotype in the decidual tissues.

Although coinhibitory marker expression in decidua has been investigated to some degree previously, our study provides a more complete analysis of these markers on T, B, and NK cell subsets, as well as the comparison between decidual compartments. The OPLS-DA model based on the expression of the coinhibitory markers PD-1, TIM-3, LAG-3, and CTLA-4 gave a clear separation between parietalis and basalis. Parietalis was associated with an increased expression of the vast majority of the investigated markers on several lymphocyte subsets. A large proportion of the T cells in both decidual compartments expressed PD-1 and TIM-3, which is in line with other studies showing that decidual T cells express higher levels of these markers compared to peripheral blood [11, 36]. PD-1, TIM-3, and LAG-3 expression on T cells was most pronounced in parietalis, which also contained a substantial CD4^+^ T cell population expressing both PD-1 and TIM-3. The ligands for PD-1, PD-L1 and PD-L2, have been shown to be expressed on decidual macrophages and stromal cells and to be functional in suppressing T cell proliferation and cytokine production [4, 36, 37]. Pregnant mice that were treated with antibodies blocking PD-1 and TIM-3 had a decreased number of live fetuses compared to the control animals, thus demonstrating the importance of these markers for a successful pregnancy outcome in mice [11]. LAG-3 has not been extensively studied in pregnancy, but it has been showed that decidual Tregs express LAG-3 [38]. When we stimulated mononuclear cells with PMA/ionomycin, we found that both CD4^+^ and CD8^+^ T cells had an IFN-*γ* response in line with that observed in PBMCs from healthy controls. It would be of interest to explore decidual T cell responsiveness to fetal and viral antigens to further investigate the role of coinhibitory markers in suppressing the decidual T cell response.

A relatively large proportion of the decidual parietalis B cells expressed PD-1, TIM-3, and CTLA-4, and the expression was consistently higher in parietalis compared to basalis. PD-1 expression has been shown to increase in B cells as a response to activation [39] and to be expressed by a subpopulation of regulatory B cells involved in hepatoma progression [40]. Although CTLA-4 has mainly been described to be expressed by T cells, it has been reported that it can also be expressed by murine B cells after T cell-induced activation [41]. CTLA-4 expression in decidual B cells has not been described previously, but we observed a distinct CTLA-4^+^ B cell population in parietalis. Direct effects of these markers on B cells are however still unclear, and the general role of B cells in fetal-maternal tolerance warrants further investigation.

MAIT cells are activated by vitamin B metabolites derived from the metabolism of certain strains of bacteria [17]. Little is known about MAIT cells in pregnancy, but we have previously shown that MAIT cells are present in decidua parietalis and have a similar capacity to respond to stimulation as peripheral MAIT cells [13]. Interestingly, we could separate basalis and parietalis in an OPLS-DA model based on data collected only on MAIT cells, demonstrating major phenotypic differences on MAIT cells between these two compartments. Basalis contained higher frequencies of MAIT cells among the CD3^+^ cells, but a larger proportion of MAIT cells in parietalis were CD4^+^ at the expense of CD8^+^ MAIT cells. Similar to the conventional T cells, the MAIT cells in parietalis displayed a more activated phenotype compared to basalis, but parietalis MAIT cells also had a significantly higher expression of the activation marker CD38. In addition, parietalis MAIT cells had higher expression of the exhaustion marker PD-1, further demonstrating their chronically activated phenotype in decidual tissues. Although the number of donors for functional analysis was few, we found that MAIT cells from both basalis and parietalis rapidly produced IFN-*γ*, GrzB, and perforin in response to *E. coli* stimulation. Our previous work on MAIT cells in decidua parietalis also showed that decidual MAIT cells produced these factors at similar proportions as peripheral blood MAIT cells [13]. PD-1 ligation has been described to hamper MAIT cell function [42], but despite the high PD-1 expression on decidual MAIT cells, their functional capacity was still intact. Our study provides the first evidence of the presence, phenotype, and functionality of MAIT cells in basalis, as well as comparison with MAIT cells from parietalis.

The physiological reasons for the differences in lymphocyte phenotypes in parietalis and basalis are not known. One potential explanation is that term basalis is more vascularized compared to parietalis, which could allow a higher influx of peripheral immune cells with a less activated phenotype, whereas the parietalis could be more static in terms of immunological composition. This could also mean that basalis is more exposed to blood-borne infectious agents, whereas parietalis mainly provides protection against mucosal pathogens as well as commensal microbiota. Another hypothesis is that labor is induced by an activation or recruitment of inflammatory immune cells to the membranes [43] and that parietalis is the site for membrane rupture. Marcellin et al. recently showed that the area of parietalis overlying the cervix displayed an altered immunological phenotype shift at term compared to the other parts of the parietalis membrane [44]. The expression of polymorphic HLA molecules was upregulated, the NK cell population shifted towards the classical cytotoxic phenotype, and immunotolerant M2 macrophages declined. We isolated lymphocytes from the entire decidua parietalis membrane and found the general leukocyte population to be dominated by the more activated phenotype. It would be interesting to investigate the T cell phenotype in parietalis tissue overlying the cervix in term decidua to examine if the effects observed by Marcellin et al. are also apparent for T cells.

With our isolation method, we aimed at procuring tissue resident immune cells as representative as possible of physiological conditions. Although the tissue was extensively washed before processing, we cannot be sure that our samples were completely devoid of contaminating peripheral blood. Since the decidua basalis is a more vascularized tissue compared to parietalis, it is also possible that the contamination was larger in the basalis samples. Also, although we did not use enzymatic digestion of the tissue, the mechanical disaggregation of the tissue could possibly have changed the expression of certain surface molecules. Another limiting factor is that our sample size was not large enough to investigate whether factors such as time of placental delivery to analysis, age of the donor, or the gestation week did influence either the isolation of immune cells or results of the analysis.

Placenta-derived stromal cells are being explored as potential candidates for cell therapy, since they are involved in regulating inflammatory immune responses [12, 45, 46]. Stromal cells and fibroblasts from different anatomical sites of the body have been shown to have distinct transcriptional patterns even after *in vitro* expansion, indicating a positional memory of stromal cells [47]. It may therefore be of importance to consider the immunological phenotype of the tissue from which the stromal cells are isolated. Differences between stromal cells isolated from decidua parietalis and basalis have not been thoroughly explored, but as evidenced by our immunophenotyping, it is possible that they have different capacities to affect immune responses.

## 5. Conclusion

We have performed a comprehensive characterization of leukocyte populations in paired samples from decidua parietalis and basalis. We found that immune cell populations differ strongly between the two investigated compartments. Decidua parietalis lymphocytes as well as MAIT cells displayed a more activated phenotype with a higher expression of coinhibitory markers than those isolated from basalis. Since decidua parietalis is the site for membrane rupture, it is interesting that the parietalis generally contained T cells of a more activated and differentiated phenotype compared to basalis. Also, these two different anatomical sites could be influenced by different factors and may play different roles during pregnancy. The results from this study may also give clues on how the stromal microenvironments at the different decidual sites may affect immune cell regulation, which potentially should be considered when selecting the source of tissue for stromal cell isolation for cell therapy.

## Supplementary Material

Supplementary Figure S1. OPLS plot based on 81 parameters, showing associations between decidual compartment and phenotypic leukocyte markers (*n*=8 − 13). Supplementary Figure S2. (A) Comparison between decidua basalis and parietalis regarding CD16 expression on CD14^+^ monocytes (*n*=11). (B) Comparison between decidua basalis and parietalis regarding CTLA-4 expression on CD4^+^ and CD8^+^ T cells (*n*=11). (C) Comparison between decidua basalis and parietalis regarding TIM-3 expression on CD56^+^ cells (*n*=11). Line in graphs depicts the median among values. Comparisons between the paired samples were made using the the non-parametric Wilcoxon test. Supplementary Table S1. Antibodies and viability dye used for flow cytometry.
