# Supplementary material for: Maternal Adaptive Immune Cells in Decidua Parietalis Display a More Activated and Coinhibitory Phenotype Compared to Decidua Basalis
Source: Stem Cells Int. 2017 Nov 29;2017:8010961. doi: 10.1155/2017/8010961 (PMC5727765; doi:10.1155/2017/8010961)
Supplement: Supplementary file 1 — Supplementary Figure S1. OPLS plot based on 81 parameters, showing associations between decidual compartment and phenotypic leukocyte markers (n=8 − 13). Supplementary Figure S2. (A) Comparison between decidua basalis and parietalis regarding CD16 expression on CD14+ monocytes (n=11). (B) Comparison between decidua basalis and parietalis regarding CTLA-4 expression on CD4+ and CD8+ T cells (n=11). (C) Comparison between decidua basalis and parietalis regarding TIM-3 expression on CD56+ cells (n=11). Line in graphs depicts the median among values. Comparisons between the paired samples were made using the the non-parametric Wilcoxon test. Supplementary Table S1. Antibodies and viability dye used for flow cytometry. [file 8010961.f1.doc]

# Maternal adaptive immune cells in decidua parietalis display a more activated and co-inhibitory phenotype compared to decidua basalis

Martin Solders, Laia Gorchs, Sebastian Gidlöf, Eleonor Tiblad, Anna-Carin Lundell and Helen Kaipe


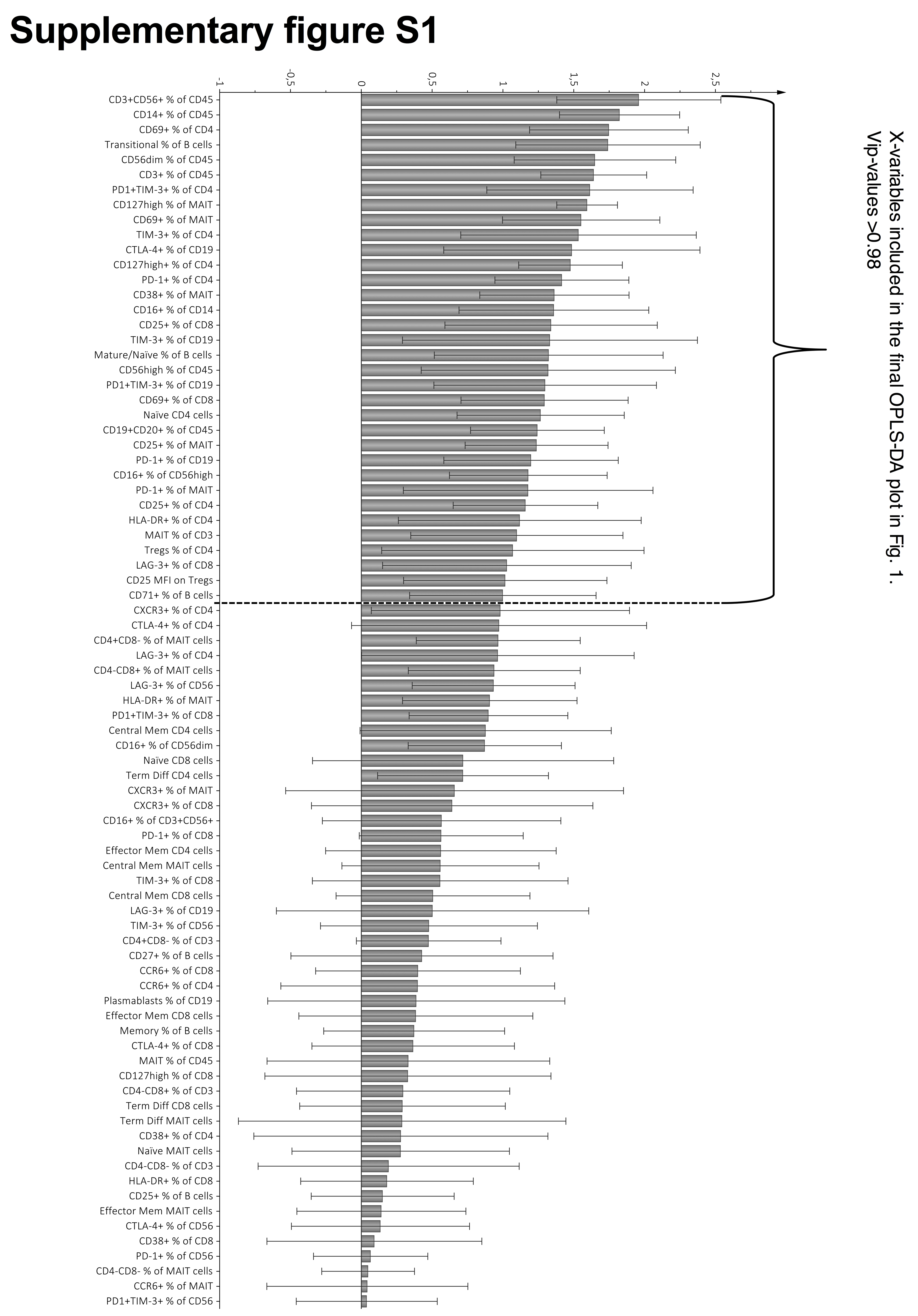
**Supplementary figure S1.** OPLS plot based on 81 parameters, showing associations between decidual compartment and phenotypic leukocyte markers (*n* = 8-13).


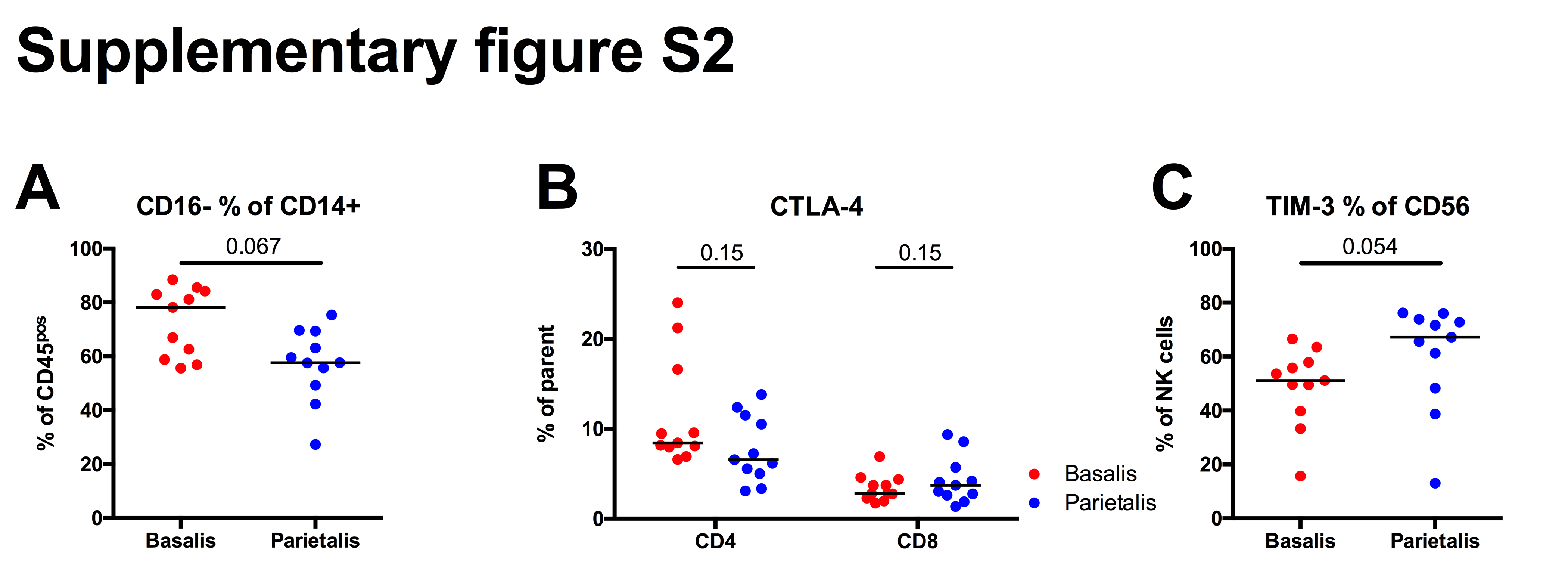
**Supplementary figure S2. (A)** Comparison between decidua basalis and parietalis regarding CD16 expression on CD14^+^ monocytes (*n* =11). **(B)** Comparison between decidua basalis and parietalis regarding CTLA-4 expression on CD4^+^ and CD8^+^ T cells (*n* =11). **(C)** Comparison between decidua basalis and parietalis regarding TIM-3 expression on CD56^+^ cells (*n* =11). Line in graphs depicts the median among values. Comparisons between the paired samples were made using the the non-parametric Wilcoxon test.

**Supplementary table S1.** Antibodies and viability dye used for flow cytometry.

| **Surface markers** | **Fluorochrome** | **Clone** | **Source** |
| --- | --- | --- | --- |
| **CD3** | V450 | UCHT1 | BD |
| **CD3** | FITC | UCHT1 | BD |
| **CD3** | PE-Cy7 | UCHT1 | BD |
| **CD4** | V500 | RPA-T4 | BD |
| **CD4** | A700 | RPA-T4 | BD |
| **CD8** | APC-Cy7 | SK1 | BD |
| **CD8** | A700 | RPA-T8 | BD |
| **CD8** | APC | SK1 | BD |
| **CD8** | PE-Cy7 | RPA-T8 | BD |
| **CD14** | FITC | MϕP9 | BD |
| **CD16** | PE | 3G8 | BD |
| **CD19** | PE-Cy7 | SJ25C1 | BD |
| **CD19** | PE-CF594 | HIB19 | BD |
| **CD20** | A700 | 2H7 | BD |
| **CD24** | FITC | NL5 | BD |
| **CD25** | Bv421 | M-A251 | BD |
| **CD25** | PE | M-A251 | BD |
| **CD27** | V500 | M-T271 | BD |
| **CD38** | Bv421 | HIT2 | BD |
| **CD45** | APC-H7 | 2D1 | BD |
| **CD45RA** | PE-Cy7 | HI100 | BD |
| **CD56** | APC | NCAM16.2 | BD |
| **CD56** | Bv510 | NCAM16.2 | BD |
| **CD69** | FITC | L78 | BD |
| **CD71** | APC-H7 | M-A712 |  |
| **CD127** | APC-A700 | R34.34 | Beckman Coulter |
| **CD152 (CTLA-4)** | FITC | A3.4H2.H12 | LS-Bio |
| **CD161** | PE | HP-3G10 | BioLegend |
| **CD183 (CXCR3)** | APC | 1C6/CXCR3 | BD |
| **CD196 (CCR6)** | FITC | G034E3 | BioLegend |
| **CD197 (CCR7)** | PE-CF594 | 150503 | BD |
| **CD223 (LAG-3)** | PE | REA351 | Miltenyi |
| **CD279 (PD-1)** | BV421 | EH12.1 | BD |
| **CD366 (TIM-3)** | APC | F38-2E2 | Miltenyi |
| **HLA-DR** | FITC | G46-6 | BD |
| **TCR Vα7.2** | APC-Cy7 | 3C10 | BioLegend |
| **-** | 7AAD | - | BD |
|  |  |  |  |
| **Intracellular markers** |  |  |  |
| **Granzyme B** | FITC | GB11 | BD |
| **IFN-γ** | PE-Cy7 | 4S.B3 | BD |
| **Perforin** | A647 | δG9 | BD |

Abbreviations; CD, Cluster of differentiation, BD, BD Biosciences (Franklin Lakes, NJ), FITC, Fluoresceinisothiocyanate, Pe-Cy7, Phycoerythrin-cyanine 7, A700, Alexa Fluor 700, APC, Allophycocyanine, PE, Phycoerythrin, Bv421, Brilliant violet 421, APC-H7, Allophycocyanine H7, APC-A700, Allophycocyanine Alexa Fluor 700, Beckman Coulter, Beckman Coulter (Fullerton, CA), BioLegend, Biolegend (San Diego, CA), PE-CF594, Phycoerythrin-CF 594, APC-Cy7, Allophycocyanine-indo tricarbocyanine, Miltenyi, Miltenyi Biotec (Bergisch Gladbach, Germany), A488, Alexa Fluor 488, A647, Alexa Fluor 647, Bv510, Brilliant violet 510, LS-Bio, LifeSpan Biosciences Inc. (Seattle, WA).
