# Supplementary material for: Maternal Adaptive Immune Cells in Decidua Parietalis Display a More Activated and Coinhibitory Phenotype Compared to Decidua Basalis
Source: Stem Cells Int. 2017 Nov 29;2017:8010961. doi: 10.1155/2017/8010961 (PMC5727765; doi:10.1155/2017/8010961)
Supplement: Supplementary file 2 [file 8010961.f2.pdf]

Maternal adaptive immune cells in decidua parietalis display a more activated and co-inhibitory phenotype compared to decidua basalis

Martin Solders, Laia Gorchs, Sebastian Gidlöf, Eleonor Tiblad, Anna-Carin Lundell and Helen Kaipe

Supplementary figure S1

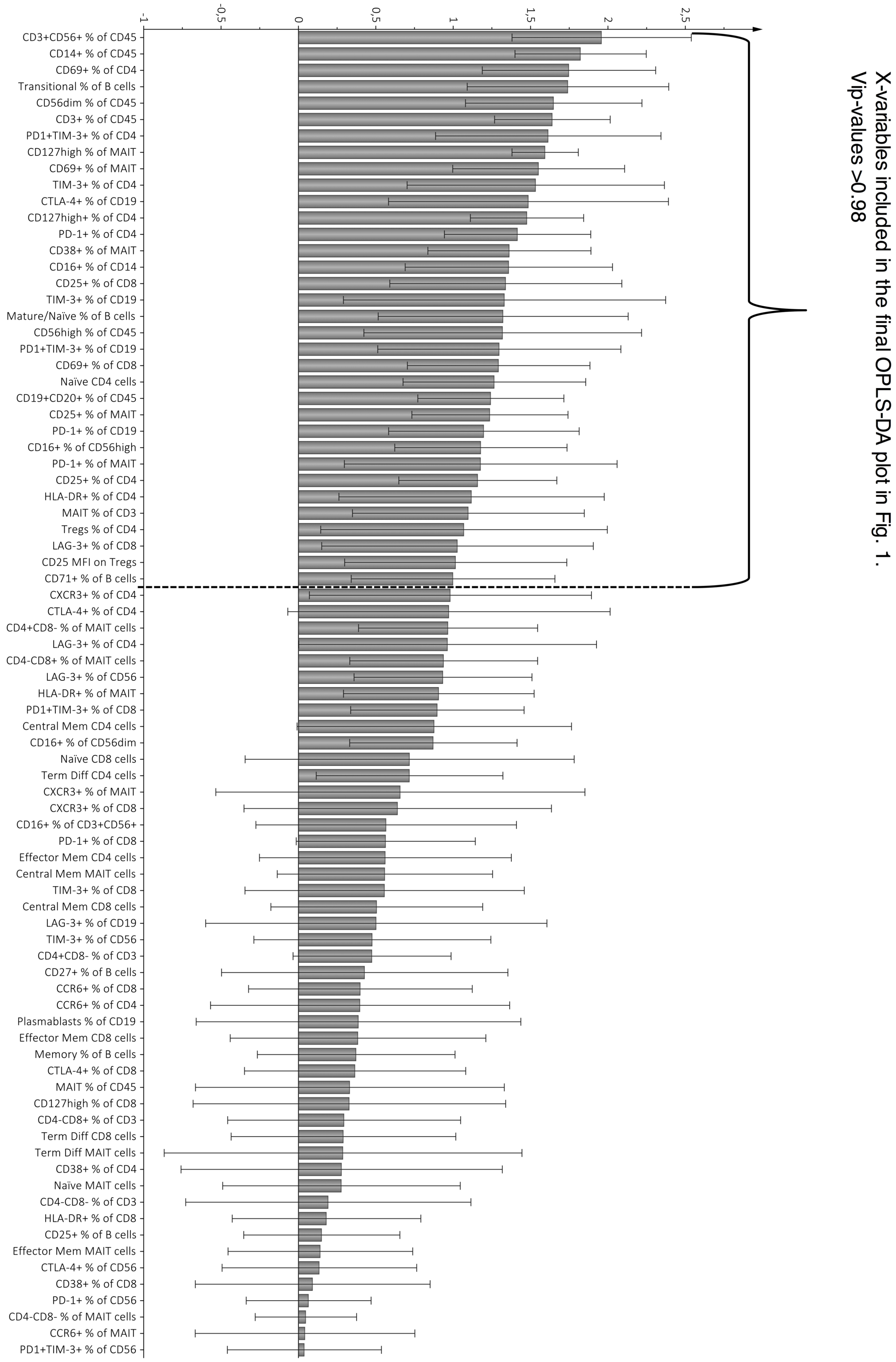

Supplementary figure S1. OPLS plot based on 81 parameters, showing associations between decidual compartment and phenotypic leukocyte markers (*n* = 8-13).

Supplementary figure S2

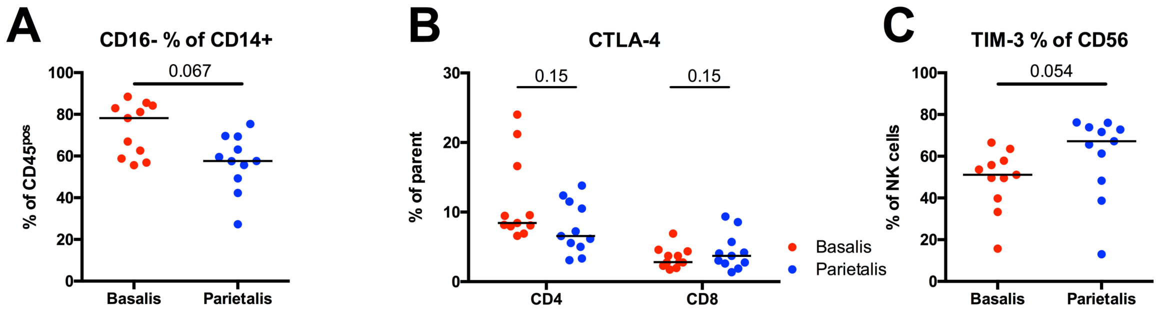

Supplementary figure S2. (A) Comparison between decidua basalis and parietalis regarding CD16 expression on $CD14^+$ monocytes (*n* =11). **(B)** Comparison between decidua basalis and parietalis regarding CTLA-4 expression on $CD4^+$ and $CD8^+$ T cells (*n* =11). **(C)** Comparison between decidua basalis and parietalis regarding TIM-3 expression on $CD56^+$ cells (*n* =11). Line in graphs depicts the median among values. Comparisons between the paired samples were made using the the non-parametric Wilcoxon test.

Supplementary table S1. Antibodies and viability dye used for flow cytometry.

Surface markers	Fluorochrome	Clone	Source
**CD3**	V450	UCHT1	BD
**CD3**	FITC	UCHT1	BD
**CD3**	PE-Cy7	UCHT1	BD
**CD4**	V500	RPA-T4	BD
**CD4**	A700	RPA-T4	BD
**CD8**	APC-Cy7	SK1	BD
**CD8**	A700	RPA-T8	BD
**CD8**	APC	SK1	BD
**CD8**	PE-Cy7	RPA-T8	BD
**CD14**	FITC	MϕP9	BD
**CD16**	PE	3G8	BD
**CD19**	PE-Cy7	SJ25C1	BD
**CD19**	PE-CF594	HIB19	BD
**CD20**	A700	2H7	BD
**CD24**	FITC	NL5	BD
**CD25**	Bv421	M-A251	BD
**CD25**	PE	M-A251	BD
**CD27**	V500	M-T271	BD
**CD38**	Bv421	HIT2	BD
**CD45**	APC-H7	2D1	BD
**CD45RA**	PE-Cy7	HI100	BD
**CD56**	APC	NCAM16.2	BD
**CD56**	Bv510	NCAM16.2	BD
**CD69**	FITC	L78	BD
**CD71**	APC-H7	M-A712	
**CD127**	APC-A700	R34.34	Beckman Coulter
**CD152 (CTLA-4)**	FITC	A3.4H2.H12	LS-Bio
**CD161**	PE	HP-3G10	BioLegend
**CD183 (CXCR3)**	APC	1C6/CXCR3	BD
**CD196 (CCR6)**	FITC	G034E3	BioLegend
**CD197 (CCR7)**	PE-CF594	150503	BD
**CD223 (LAG-3)**	PE	REA351	Miltenyi
**CD279 (PD-1)**	BV421	EH12.1	BD
**CD366 (TIM-3)**	APC	F38-2E2	Miltenyi
**HLA-DR**	FITC	G46-6	BD
**TCR Vα7.2**	APC-Cy7	3C10	BioLegend
**-**	7AAD	-	BD

**Intracellular markers**			
**Granzyme B**	FITC	GB11	BD
**IFN-γ**	PE-Cy7	4S.B3	BD
**Perforin**	A647	δG9	BD

Abbreviations; CD, Cluster of differentiation, BD, BD Biosciences (Franklin Lakes, NJ), FITC, Fluoresceinisothiocyanate, Pe-Cy7, Phycoerythrin-cyanine 7, A700, Alexa Fluor 700, APC, Allophycocyanine, PE, Phycoerythrin, Bv421, Brilliant violet 421, APC-H7, Allophycocyanine H7, APC-A700, Allophycocyanine Alexa Fluor 700, Beckman Coulter, Beckman Coulter (Fullerton, CA), BioLegend, Biolegend (San Diego, CA), PE-CF594, Phycoerythrin-CF 594, APC-Cy7, Allophycocyanine-indo tricarbocyanine, Miltenyi, Miltenyi Biotec (Bergisch Gladbach, Germany), A488, Alexa Fluor 488, A647, Alexa Fluor 647, Bv510, Brilliant violet 510, LS-Bio, LifeSpan Biosciences Inc. (Seattle, WA).
